# Mechanistic modelling of interventions against spread of livestock-associated methicillin-resistant *Staphylococcus aureus* (LA-MRSA) within a Danish farrow-to-finish pig herd

**DOI:** 10.1371/journal.pone.0200563

**Published:** 2018-07-12

**Authors:** Anna Irene Vedel Sørensen, Thomas Rosendal, Stefan Widgren, Tariq Halasa

**Affiliations:** 1 Division for Diagnostics and Scientific Advice, National Veterinary Institute, Technical University of Denmark, Lyngby, Denmark; 2 Department of Disease Control and Epidemiology, National Veterinary Institute, Uppsala, Sweden; Wageningen Universiteit en Researchcentrum IMARES, NETHERLANDS

## Abstract

Knowledge on successful interventions against livestock-associated methicillin-resistant *Staphylococcus aureus* (LA-MRSA) within pig herds is sparse. In situations like this, a mechanistic simulation model can be a valuable tool for assessing the effect of potential intervention strategies, and prioritising which should be tested in the field. We have simulated on-farm interventions in a farrow-to-finish pig herd, with a previously published LA-MRSA spread model, within four different areas: 1) Reduced antimicrobial consumption, 2) Reduced number of pigs within each section, 3) Reduced mixing of pigs, and 4) Improved internal biosecurity. To model a decrease in the selective pressure, the transmission rates were reduced after LA-MRSA had become fully established within a herd, which resulted in a marked decrease in the prevalence within all stable units. However, LA-MRSA rarely disappeared completely from the herd; this was only observed in scenarios where the transmission rates were reduced to ≤ 30% of the original level. While changes in antimicrobial consumption patterns might be a very important step towards reducing the spread of LA-MRSA, the simulation results indicate that it may need to be paired with other preventive or intervention measures. Reducing the number of pigs within each section, reducing mixing of pigs, or improving internal biosecurity after LA-MRSA had become established within the herd only resulted in marginal changes in the median prevalence within the herd. However, these factors might be important in relation to being able to achieve or maintain a low level of antimicrobial consumption, and thus still indirectly influence the LA-MRSA prevalence within the herd. The results of a sensitivity analysis indicated the assumptions regarding the existence of pigs persistently shedding MRSA have a noticeable influence on the model results. The assumptions regarding transmission from sow to offspring at the day of birth also had a considerable influence on the MRSA prevalence within the farrowing unit but did not cause any marked changes in the simulated effect of interventions. Effects might differ between different farm types contaminated in different levels and this simulation study highlights a strong need for more knowledge from on-farm trials.

## Introduction

*Staphylococcus aureus* is an opportunistic pathogen capable of causing a wide-range of diseases in humans and animals [1]. In 2005, findings of livestock-associated methicillin-resistant *Staphylococcus aureus* (LA-MRSA) were reported for the first time in France and the Netherlands [2,3], and since then LA-MRSA has been detected in the pig population in many European countries [1].

The majority of LA-MRSA strains are resistant to tetracyclines [4] and use of these compounds is therefore expected to select for LA-MRSA. In a longitudinal study, where transmission rates of LA-MRSA between pigs were estimated both with and without the use of risk-antimicrobials (beta-lactams and tetracyclines), these were significantly different from each other [5], and in several studies group treatment with antimicrobials has been identified as a risk factor for pig farms becoming LA-MRSA contaminated [6–8]. Also, in an intervention study, where use of antimicrobials was reduced by 44%, this was associated with declining MRSA prevalence in pigs [9]. Thus, changing antimicrobial consumption patterns on the farms can be considered a relevant area of intervention.

LA-MRSA has been detected in high levels in air within stable units [10], and consequently pigs are exposed to LA-MRSA both through bacteria bound to dust particles suspended in the air, and through direct contact with their pen mates. Since the LA-MRSA contamination of the air is assumed to originate from pigs shedding LA-MRSA, a reduction in the number of pigs within a stable section might lead to decreased exposure, both through decreased concentrations in the air and through decreased direct contact to other pigs, provided that the within-pen stocking-density is also reduced. The number of direct contact events with other pigs during an animal’s lifespan is dependent both on the stocking-density in the pens, and on how often mixing between pigs in different pens or batches occur. Both factors have been identified as risk factors for spread of other infectious agents [11,12].

In addition to in the air within stables, LA-MRSA has been detected in many different parts of the farm environment, including in dust, feed, faeces, and boot swabs of the service alley on contaminated farms [10]. Therefore, farm workers and equipment are also potential sources of spread of LA-MRSA between sections or stable units within the farm. Some units are more work intensive than others, e.g. the farrowing unit, and the work will involve more direct interaction between humans and pigs. Improved internal biosecurity, e.g. improved hand hygiene, change of boots between stables, fixed working order, having equipment dedicated to each unit etc., may reduce this spread.

On-farm studies showing successful interventions against spread of LA-MRSA, which do not involve emptying the farm and culling all animals, are sparse. Most of these have focused on the use of disinfectants, but the scope, study design, disinfection procedure and type of disinfectant applied varied, and so did the results. In general, it has been shown possible to remove LA-MRSA entirely through disinfection in the absence of animals [13,14], or obtain a reduction in LA-MRSA levels in the air and bedding materials, when repeatedly applying disinfectant in the presence of LA-MRSA positive animals [15]. Other attempts at reducing the LA-MRSA contamination within farms, includes sow washing, where the original strain was detected in 64% of the animals again after washing [16], and use of an air cleaning system consisting of an air washer and a UV-irradiation system, which led to significantly reduced concentrations of LA-MRSA in the stable air [17].

In situations where the knowledge on successful interventions is limited, a mechanistic simulation model can be a valuable tool for assessing the effect of potential intervention strategies, and prioritising which one should be tested on real farms. One of the main challenges, when modelling spread of LA-MRSA is that the dynamics of infection in pigs are not clear, and assumptions regarding the existence of both intermittent shedders (IS) and persistent shedders (PS) might have a major impact on the results. In this paper, we use a previously published mechanistic individual-based model for spread of LA-MRSA within a pig herd [18] for simulating the outcome of implementing on-farm interventions within four different areas: 1) Reduced antimicrobial consumption, 2) Reduced number of pigs within each section, 3) Reduced mixing of pigs, and 4) Improved internal biosecurity. Using the Danish situation as an example, where LA-MRSA was isolated from 88% of 57 randomly selected pig herds tested in Denmark [19], we assume that LA-MRSA has already become fully established within the herd and reached a steady state prevalence in all farm units before the interventions are initiated. The aims of the study were to: 1) Assess the effect of the possible intervention strategies mentioned above and evaluate if it is possible to clear a farm from LA-MRSA, once it has become established by lowering the transmission, and 2) Assess the impact of assumptions and parameters on model predictions.

## Materials and methods

### Simulation model

All simulations and data analyses were carried out in R version 3.2.2 –“Fire Safety” [20]. The model used for the simulations is a mechanistic, stochastic, individual-based model with discrete time-steps of one day. All simulation scenarios were run for 500 iterations, except in the sensitivity analysis, where some simulations were run with 100 iterations as explained below in section: ‘Sensitivity analysis’. The model consists of two main units, a herd model of a farrow-to-finish pig herd and an epidemic model for LA-MRSA. Both are briefly described below and a more detailed description of the full model can be found in Sørensen et al., 2017 [18], including a link to the model R-code: https://github.com/anvso/DTU-model.

### Herd model

The model was designed to represent a typical Danish medium-sized farrow-to-finish herd (~ 500 sows, annual production: ~15,400 slaughter pigs). It was assumed that the herd used weekly batch production with 100% artificial insemination, and replacement gilts were purchased from other herds. The main processes in the model included: insemination, farrowing, slaughter, death/culling, re-insemination and use of two-step nurse sows. The farm consisted of five different stable units: the mating unit, the gestation unit, the farrowing unit, the weaner unit and the finisher unit.

### Epidemic model

The epidemic model used for LA-MRSA was an SIS-model with two different infectious stages, since it was assumed that a pig could either be susceptible to LA-MRSA, or be an intermittent or persistent shedder of LA-MRSA. It was assumed that, as in humans, IS and PS formed two distinct groups [21], and therefore a pig could not go directly from being an IS to becoming a PS. Whether a pig became a PS was modelled to depend both on host-related factors and the degree of exposure to LA-MRSA. This was implemented by only assigning a certain fraction of the pigs the potential to become PS, with a probability of becoming PS upon exposure that changed depending on the prevalence within the section where the pig was housed being above or below a given limit [18]. The duration of shedding for IS varied from 1–26 days. The routes of transmission in the model included: within-pen, between-pen, between-section and between-stable transmission [18]. A special route of transmission from sows to new-born piglets on the day of farrowing was included in the model in order to also account for perinatal transmission [18].

### Interventions simulated

To allow enough time for LA-MRSA to become established in pigs within all stable units, the interventions were not initiated until 180 days after LA-MRSA had been introduced, reflecting the time needed for LA-MRSA to reach an endemic state. It was assumed that this was a single introduction, i.e. all gilts entering the model later were assumed to be negative. This is a relevant approach to testing interventions that could be useful if implemented in the endemic state currently found in Danish pig population. All scenarios were simulated with three different sets of transmission rates, referred to as ‘low’, ‘medium’ and ‘high’, except the scenarios related to reduced antimicrobial consumption, where the high rates were used as a baseline before intervention. The ‘low’ set of transmission rates were intended to represent a scenario with no use of beta-lactams or tetracyclines (rates based on [5]), whereas ‘high’ transmission represented a situation with high antimicrobial consumption (rates based on [5]). The ‘medium’ scenario represented a situation between the two extremes (rates were based on averages of ‘low’ and ‘high’) [18].

### Reduced antimicrobial consumption

Changes in the antimicrobial consumption patterns, which lead to a decrease in the use of compounds selecting for LA-MRSA, are expected to decrease the rate of LA-MRSA transmission between pigs. To investigate if it was possible to clear a herd from LA-MRSA by decreasing transmission to a sufficiently low level, all the transmissions rates used when assuming ‘high’ transmission, were reduced by 10% - 90% in steps of 10% each.

### Reduced number of pigs within each section

In Denmark, it is common for farmers to sell pigs either immediately after weaning (weight ~7 kg) or after the nursery phase (weight ~30 kg), and therefore scenarios were specified with reductions in the number of pigs in each section within these two age groups, where we assumed that the farmer started selling pigs and gradually increased the proportion of every batch sold by 5%-steps every 6^th^ month. The overall stocking density within a section was reduced, by either: 1) utilising less of the pens available within the section, or 2) reducing the number of pigs within each pen (reduced stocking density). It was assumed that a reduction in stocking density also would affect the transmission rate due to decreased contact rate, and thus the transmission rates were reduced stepwise with the same relative reduction per step as the relative reduction in density.

### Reduced mixing

In the simulated herd, it was assumed that the farmer was using batch production i.e. in principle all-in/all-out on the section level. However, animals from different batches might in some cases be mixed. Regularly, some sows will be moved from one batch to another, either because of reproductive failure or because of being used as nurse sows (foster dams) for piglets born by sows in other batches. In a survey from 2016, 63% and 52% of the interviewed Danish pig herd owners, who used batch production, had a buffer section in their weaner or finisher unit, respectively (S1 Table). Therefore, both the weaner and the finisher units were assumed to contain a buffer section for slower growing pigs that needed extra time in the unit before being ready to be moved to the finisher stable or before being sent for slaughter. It was assumed that the leftover pigs in the weaner unit could spend up to three weeks in the buffer section before being moved to the finisher unit and that these pigs would therefore be mixed with weaners from other batches. Mixing of pigs in the buffer section in the finisher unit was considered to only be of little importance, since pigs will not return from the buffer section, but instead be sent directly to slaughter from here. Mixing of pigs from different litters is common in Danish pig production herds, where pigs are frequently sorted according to size and assigned new pen mates when they are moved from one stable unit to another. In the baseline scenario, it was assumed that the pigs were sorted and assigned new pen mates at least twice: first when entering the weaner unit, and later when being moved from the weaner to the finisher unit. In practise, this was implemented in the model as random mixing at transition. In the present study, reduced mixing was simulated in three different ways: 1) No use of buffer sections and thus no possibility of mixing between pigs belonging to different batches, 2) No use of buffer stables along with reduced mixing (the pigs in each weaner pen were distributed in two pens, when being moved to the finisher unit. As a result, these pigs only received new pen mates once, at entry in the weaner unit, when two litters would be put into one pen together), 3) Keeping pigs from different litters separated all the way from farrowing and to slaughter. In practice, this also meant that the number of pigs within each pen was reduced considerably, because in the baseline scenario the maximum number of pigs per pen would be 30 in the weaner unit and 15 in finisher unit.

### Improved internal biosecurity

The effect of increased internal biosecurity was modelled as a reduction in the transmission between sections, between stable units or both, and included the extreme cases of no between-section and/or between-stable transmission.

### Sensitivity analysis

Many of the parameters used in the infection model originate from one study [5], and thus are subject to considerable uncertainty. In the first part of the sensitivity analysis, the effect of changes in within-pen and between-pen transmission rates relative to each other was investigated. This was intended to highlight which changes in model parameters that have the biggest impact on the outcome and therefore potential focus areas for intervention. Within-pen and between-pen transmission parameters were independently scaled from 0.3–1.1 times the baseline value in steps of 0.10, while the other parameters were kept constant. In the sensitivity analysis, the uncertainty around these parameters was not included, and therefore simulations only required 100 iterations to generate stable estimates. Spread between-section and between-stable units were set to a fixed proportion of the between-pen spread, and therefore no separate sensitivity analysis was conducted on these. To assess the influence of intervening in one stable unit only, the within-pen and between-pen transmission rates were also changed individually in one unit at a time, where both rates were scaled with the same factor in each step.

The presence of pigs persistently shedding LA-MRSA is expected to have a considerable influence on the outcome of the intervention scenarios, and therefore the influence of the presence or absence of these was assessed in the second part of the sensitivity analysis. The assumptions regarding the probability of transmission from sow to offspring on the day of birth might also influence the interventions modelled and therefore this parameter was also subjected to sensitivity analysis. The effect of using values corresponding to 0%, 25%, 50 and 75% of the probabilities used in the standard parameterisation was investigated. The sensitivity analysis was conducted with only one set of transmission rates (‘high’ transmission).

## Results

### Reduced antimicrobial consumption

**Fig 1:**
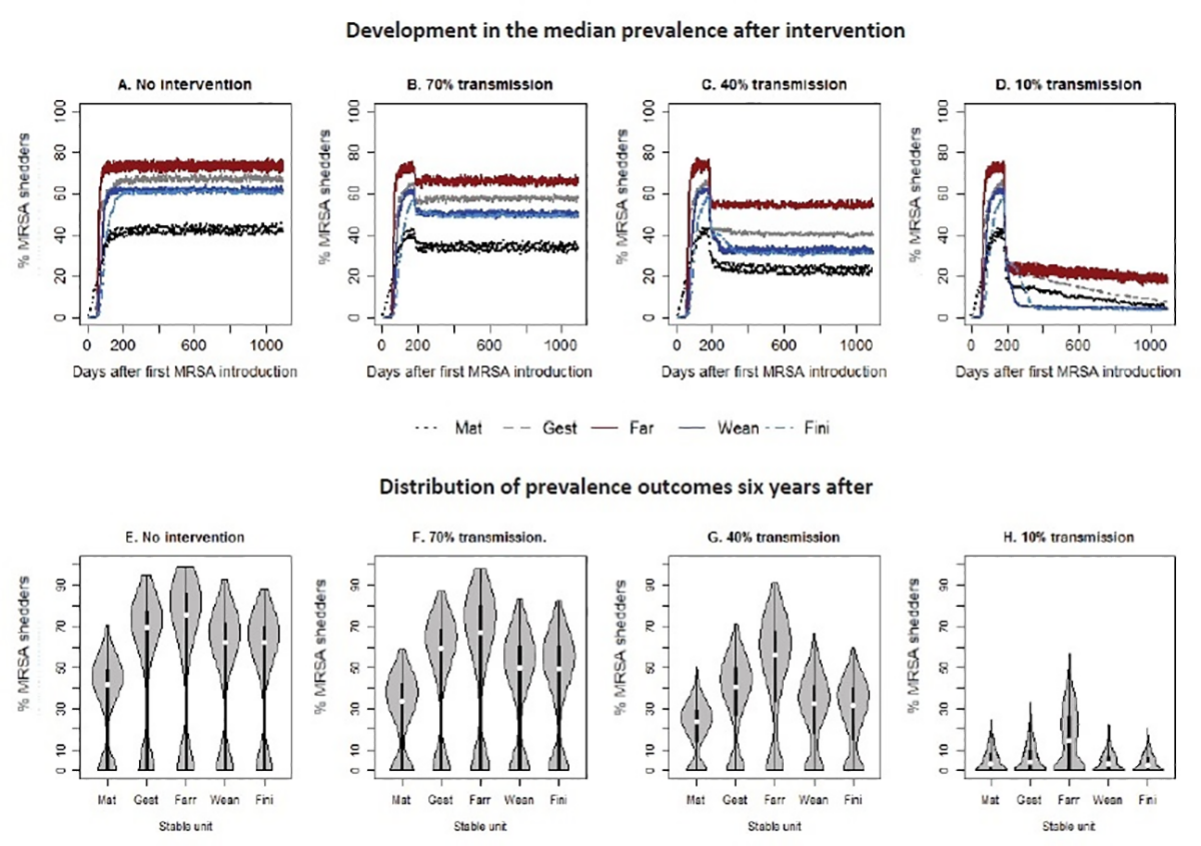
Reduced transmission. Transmission was reduced 180 days after MRSA had been introduced. The percentages refer to the proportion of the ‘high’ set of transmission rates, the set of rates used after intervention at day 180 corresponds to. Mat = mating unit, Gest = gestation unit, Farr = farrowing unit, Wean = weaner unit, Fini = finisher unit.

The median prevalence within the stable units over time decreased immediately after the reduction in transmission rates had been implemented, and then stabilised at a lower level, which depended on the proportion of reduction implemented (Fig 1A–1D and S1 Fig). Violin plots were used to illustrate the variation in the outcome of different iterations (Fig 1E–1H and S2 Fig). Generally, a bimodal distribution was observed with one proportion of the simulated prevalences clustering just above zero, and the more the transmission rates were reduced, the more iterations resulted in a prevalence of zero or just above zero (Fig 1E–1H and S2 Fig). Complete fade-out of LA-MRSA resulting from the introduced reduction in the transmission rate was only observed in the scenarios where the transmission rate was reduced to less than 30% of the initial level, and still this was a rare event (0.2%, 0.4% and 2.4% of iterations, when the transmission rates were reduced to 30%, 20% and 10% of the initial level, respectively (S2 Fig)).

### Reduced number of pigs within each section

**Fig 2:**
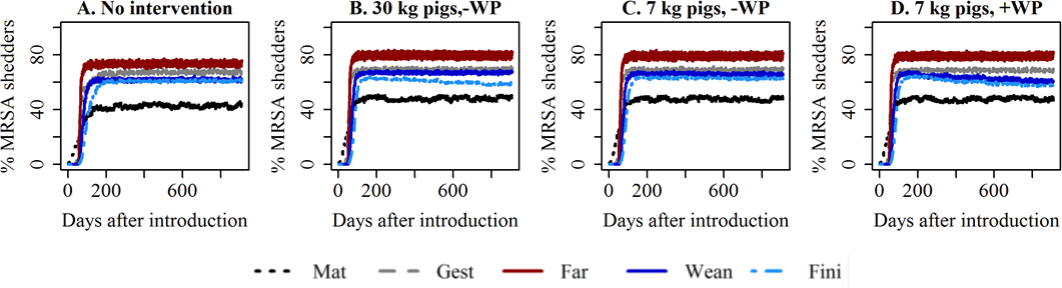
Reduced number of pigs within each section. Development in the median number and prevalence of MRSA shedders over time. High transmission. Mat = mating unit, Gest = gestation unit, Far = farrowing unit, Wean = weaner unit, Fin = finisher unit. The number of pigs within the relevant unit was gradually reduced by 5% every 6^th^ month. 7 kg pigs/30 kg pigs refer to if the pigs are sold just after weaning (7 kg) or not until they reach approximately 30 kg, which also is the point, where they normally would be moved from the weaner to the finisher unit. -/+ WP reduction refers if the within-pen density has also been reduced, or if some pens are just empty–the overall within-room density will be the same in both scenarios.

Reducing the number of pigs within each section in either way had only a marginal effect on the development in the simulated median prevalence over time, when assuming ‘high’ transmission (Fig 2), since a major effect was only observed, when enough time had elapsed for the number of pigs within each section to be reduced to level, that probably not will be realistic for farmers (>10% reduction). In the scenarios, where ‘low’ or ‘medium’ transmission was assumed, similar results were obtained (S3 Fig). There was slightly more effect, when the number of pigs was reduced within each pen and not only the number of pens in use within the section.

### Reduced mixing

**Fig 3:**
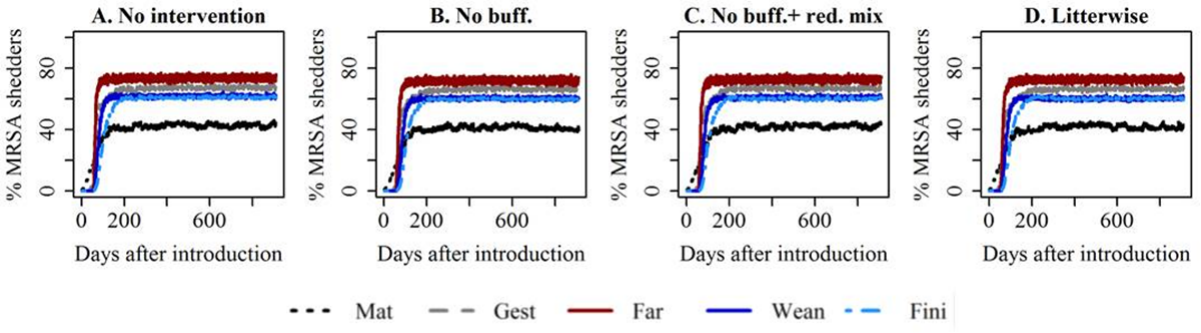
Reduced mixing. Development in the median prevalence of MRSA shedders over time. High transmission. Transmission was reduced 180 days after MRSA had been introduced. Mat = mating unit, Gest = gestation unit, Far = farrowing unit, Wean = weaner unit, Fin = finisher unit. No buff = no use of buffer sections. Red. Mix = Reduced mixing–two litters are put into one pen together in the weaners unit, instead of random mixing of piglets. Litterwise = weaners and finishers are only sharing pens with pigs from the same litter as themselves.

With the current parameterisation of the model, no effect was observed in any of the scenarios with reduced mixing, no matter if high (Fig 3), medium or low (S4 Fig) transmission was assumed.

### Improved internal biosecurity

**Fig 4:**
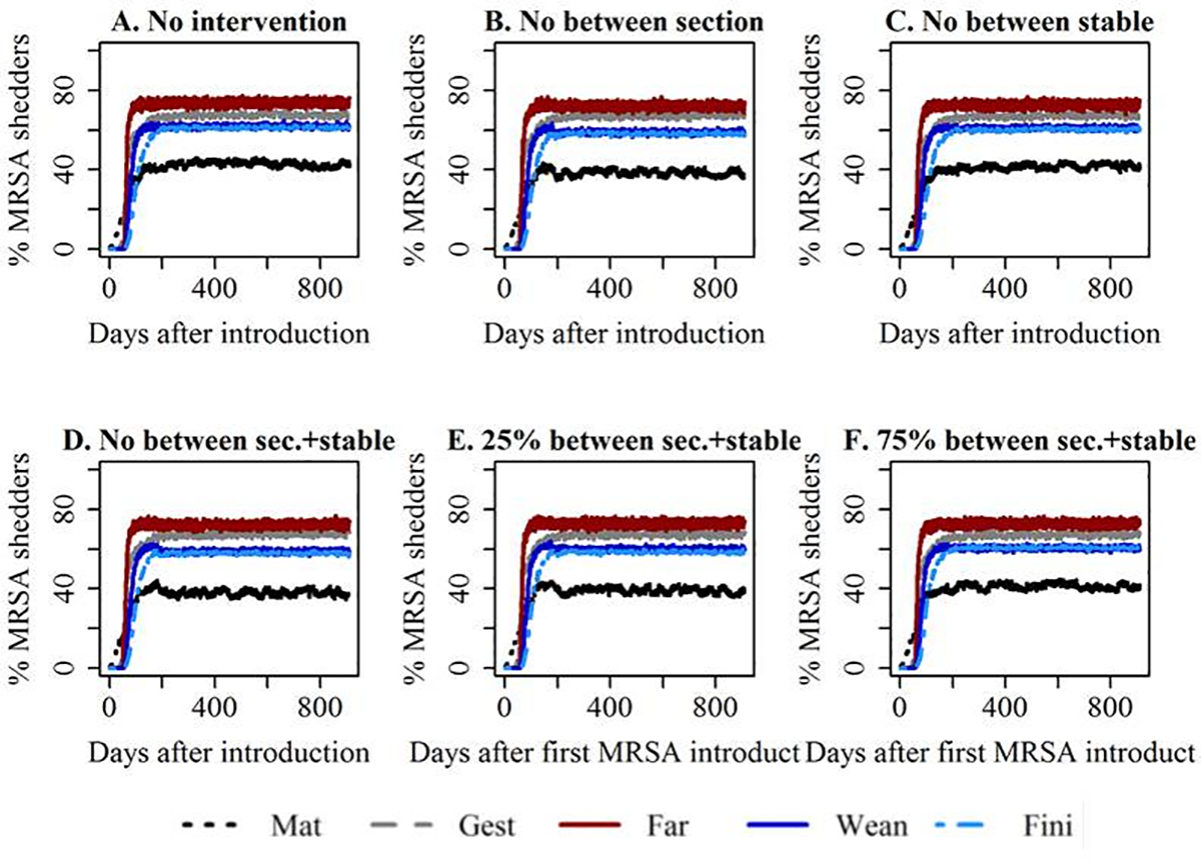
Improved internal biosecurity. Development in the median prevalence of MRSA shedders over time. High transmission. Transmission was reduced 180 days after MRSA had been introduced. Mat = mating unit, Gest = gestation unit, Far = farrowing unit, Wean = weaner unit, Fin = finisher unit. Panel B-D illustrates the extreme cases of completely eliminating between compartment transmission, whereas E and F illustrate the influence of a reduction of the transmission between sections and stables to 25% (E) or 75% of the original value (F).

Reducing transmission between sections had no noticeable effect, when LA-MRSA had already become established within the herd (Fig 4). However, when ‘low’ transmission rates were used, a small temporary drop in the prevalence was observed immediately after intervention when it was assumed that the spread between section and between stables had been reduced to 25% of the initial level (S5 Fig).

### Sensitivity analysis

**Fig 5:**
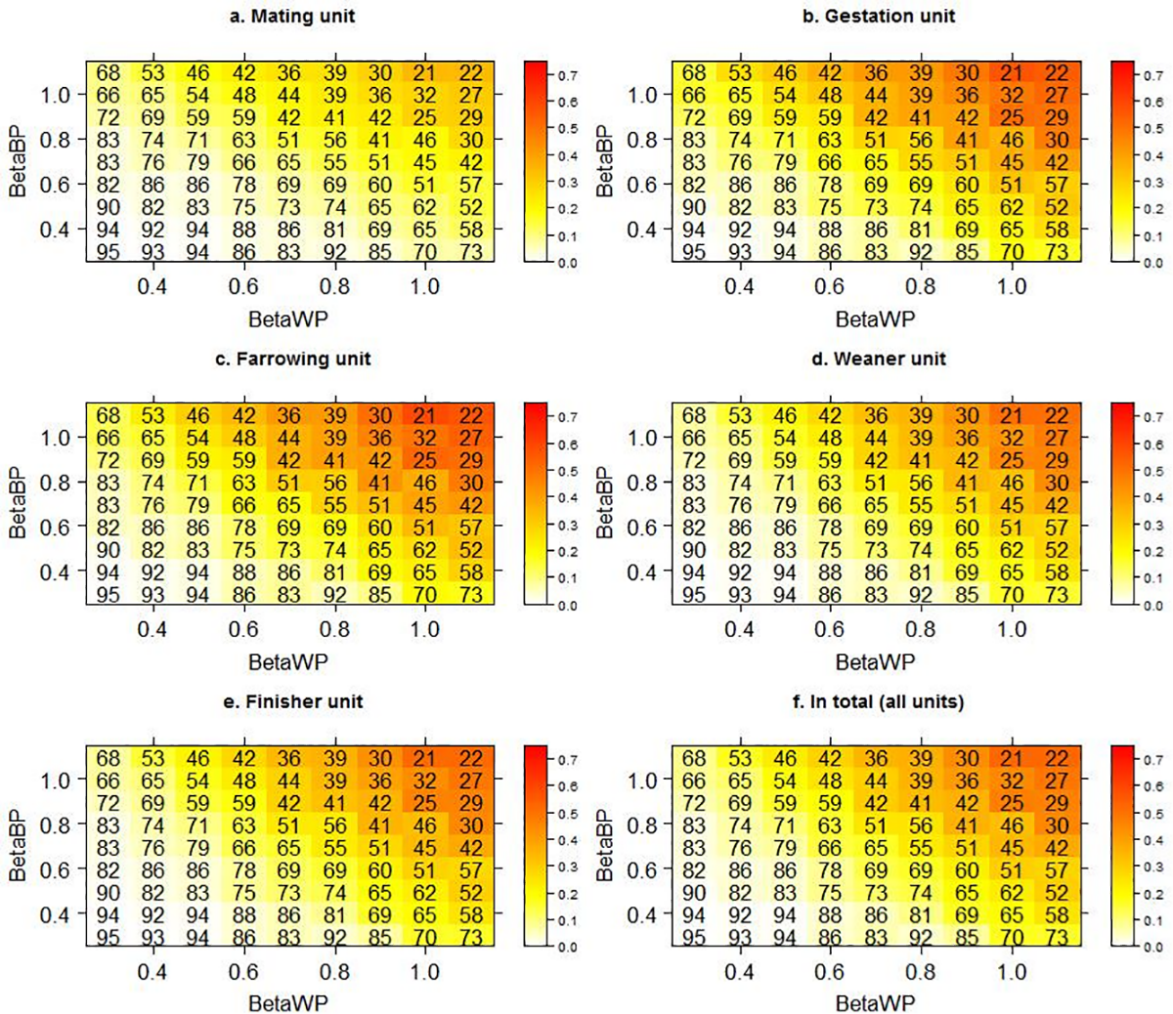
Mean prevalence in the stable units following changes in the within-pen and the between-pen transmission rate. The colour intensity represents the mean prevalence. The proportion of iterations where MRSA did not become established has been printed on each square. BetaBP = between-pen transmission rate, scaled as indicated. BetaWP = within-pen transmission rate, scaled as indicated.

The mean prevalence values after stabilisation in the five different stable units as well as the overall mean prevalence within the herd for different combinations of scaling of the transmission rates for within-pen transmission and between-pen transmission are illustrated on Fig 5. The proportion of iterations where LA-MRSA faded out was the same for all units, since following introduction LA-MRSA either faded out in all units of the farm or became established within all units. In general, the highest prevalence was observed within the farrowing unit and the lowest within the mating unit. The changes in mean prevalence followed the same overall pattern within all stable units.

Gradually changing the parameter values for all transmission rates within one stable unit at a time resulted in a gradually changing mean prevalence within the stable unit, where the changes were applied. The changes did not markedly influence the mean prevalence in the other stable units, as the prevalence consistently remained lowest in the mating unit and highest in the farrowing unit (S6 Fig).

The influence of our assumptions about the existence of persistent shedders (PS) was assessed by running selected scenarios with no PS (S7 Fig). In general, the median prevalence stabilised at a lower level, when there was no PS, but only to a lesser degree in the farrowing unit, where there is a constant supply of new susceptible piglets and a high sow-to-offspring transmission (S7B Fig). The presence of PS in the model limited the possible decrease in prevalence following intervention. For reduced mixing and improved internal biosecurity, no effect was visible when simulating with the standard parameterisation that included the presence of PS, however, when running the scenario, where the pigs were kept together with their litter without PS, there was a marginal drop in the median prevalence within the weaner and finisher units (S7E Fig). When transmission between stables and sections were reduced by 50% in a scenario without PS, a small decrease was observed in all units immediately after intervention, except in the gestation unit (which is not separated into sections due to loose housing of the sows in larger groups), and in the farrowing unit (where sow to offspring transmission quickly generates new MRSA shedders) (S7F Fig). However, the effect observed was still far too small to be of any practical importance for field intervention.

The assumption regarding transmission from sow to offspring on the day of birth had a considerable influence on the prevalence within the farrowing unit (S8 Fig) but did not markedly alter the effect of any of the simulated interventions (S9 Fig).

## Discussion

Reducing the transmission rates after LA-MRSA had become fully established within a herd to simulate a reduction in the selective pressure, resulted in a marked decrease in the prevalence within all stable units. However, LA-MRSA rarely disappeared completely from the herd and only in scenarios where the transmission rates were reduced to ≤ 30% of the original level. A reduction to ~40% of the original level corresponds to the transmission rates observed in a transmission study in the Netherlands, when no beta-lactams or tetracyclines were used [5], but it must be expected that multiple factors related to management and the environment would affect transmission, and hence it remains unknown how large a reduction would be realistic. It has however been suggested that a reduction in the overall use of antimicrobials and especially those agents which co-select for LA-MRSA, might not result in a rapid decline in the occurrence of LA-MRSA; the effect will depend on the fitness cost of methicillin resistance for LA-MRSA and the impact of management and treatment procedures implemented to replace the current procedures [4]. Additionally, one could speculate that the high stability of tetracyclines and their ability to persist in the environment [22], might play a role.

In an intervention study of 36 Dutch pig farms, where the antimicrobial use decreased by 44% during the 18-month study, this decline was associated with a decreasing MRSA prevalence in pigs, despite tetracyclines and penicillins remaining the two most used drug types during the study period [9]. The observed decrease in prevalence did not occur as fast as those resulting from abrupt reduction of the transmission rates as in the present study, where an immediate rather than gradual reduction in the use of antimicrobials was assumed. Additionally, we assumed that the use of tetracyclines and penicillins would also be reduced. A reduction in transmission could also represent the effect of reducing the concentration of LA-MRSA in the air and the environment through for instance use of a disinfectant powder.

Reducing the number of pigs within each section after LA-MRSA had become established within the herd only resulted in marginal changes in the median prevalence within the herd, if the reduction should be kept within a range that is assumed to be economically feasible for the farmer (5–10%). These changes could all be attributed to the reduction in transmission rate implemented, rather than directly to the reduced number of animals within the section or pen. For density-dependent transmission, the transmission rates depend on the population size, and the estimate of transmission rate decrease with decreasing stocking-density are difficult to assess.

In the present study, no effect of modelling reduced mixing of pigs was observed. An investigation of the LA-MRSA status of piglets at the time of intervention, when the prevalence in the herd had stabilized at a high level, revealed that most piglets and litters were already LA-MRSA positive. The effect of reduced mixing between litters could intuitively not be observed when the majority of piglets were already shedders. However, even when applying lower transmission at day-one in the piglets’ life in the sensitivity analysis, there was no apparent effect.

No environmental carryover effect was included in LA-MRSA model used, i.e. we assumed perfect disinfection between batches [18]. We also assumed that LA-MRSA could quite easily be spread between different compartments on the farm, if internal biosecurity procedures to avoid this were not practiced. LA-MRSA isolates originating from pig farms have been shown to be able to form robust biofilm under lab conditions [23], and thus may be able to survive on equipment for a long time. In the present study, no direct effect was observed as all units on the farm had already been contaminated, but this might still be important as a preventive measure in situations where LA-MRSA has not been introduced or in relation to keeping antimicrobial consumption low.

From the results of the sensitivity analysis, it became clear that our assumption regarding the existence of pigs persistently shedding LA-MRSA had a considerable influence on the results of the simulated interventions. The sensitivity analysis also revealed, that our assumption regarding transmission from sow to offspring at the day of birth, had a considerable influence on the general prevalence within the farrowing unit (S8 Fig), but not much influence on the effect of the simulated interventions (S9 Fig). The association between sow LA-MRSA status and the probability of piglets testing LA-MRSA positive have been confirmed in several studies [24–26], where the proportion of positive piglets in the days after farrowing were very different. We therefore expect the transmission on the day of birth might be dependent on the general infectious pressure on the farm, and therefore all the situations included in the sensitivity analysis could potentially be of practical relevance. Strongly decreased transmission at the day of birth could also represent the use of caesarean sections, as might be used to generate gnotobiotic pigs in nucleus breeding herds, e.g. if wanting to start a new LA-MRSA free production [27].

With the current parameterisation of the model, prevalences were in general highest within the farrowing unit, and lowest within the mating unit (S6 Fig), and thus the farrowing unit seems to be the area with the most potential for intervention. Also, changes within this unit seemed to have the most effect on the prevalence within the other units (S6 Fig).

When assessing the feasibility of the suggested interventions, practical and economic implications for the farmers should be considered, including any effects on health, welfare and growth rate of the pigs. Reducing antimicrobial consumption might be challenging, but the implementation of herd-specific interventions have in some cases been shown to reduce the use of antimicrobials without negative impact on overall economic and technical performance [28]. However, both the current antimicrobial consumption patterns and the reduction, that is possible to obtain, might of course vary considerable between farms, depending on management and current disease problems.

Also, while no direct effect of reducing the number of pigs within each section, reducing mixing or improving internal biosecurity were observed in the simulations conducted with the current parameterisation of the model, these might all be important in relation to spread of other diseases, and consequently the antimicrobial consumption within the herd. Additionally, reducing the stocking density might improve animal welfare.

Preferably, there should be multiple benefits of the interventions, which require an investment from the farmer; either these should be a step toward not only reducing the occurrence of LA-MRSA, but also the occurrence of antimicrobial resistance in general or other problematic resistant bacteria such as extended-spectrum beta-lactamase producing bacteria (ESBL), or have a preventive effect on spread of disease within the herd in general. Also, it is crucial to obtain more knowledge on how to avoid MRSA being introduced or reintroduced in the herd.

Based on the results obtained from the present simulation study, it is unlikely that a highly contaminated farm can clear itself completely from LA-MRSA by only implementing interventions, which decrease transmission, e.g. reduced use of antimicrobials and zinc. However, this intervention did result in a marked decrease in the within-herd prevalence and might play an important role in preventing LA-MRSA in becoming established in a naïve herd. Additionally, an association between human LA-MRSA carriage and air concentrations of LA-MRSA have been reported [29,30], and since one would assume that a markedly reduced prevalence of shedders within the pig barn would also lead to reduced concentration of LA-MRSA in the air within the pig barn, it could be speculated, that such a reduction might also be of value in relation to reducing spread of LA-MRSA into the general population.

It is important to keep in mind, that LA-MRSA has been found in organic [31] and antimicrobial-free herds [25], albeit much less frequently compared to in conventional herds (6% positive Danish organic herds in 2015 vs 68% positive Danish conventional herds in 2014). Therefore, while changes in antimicrobial consumption patterns might be an important step towards reducing the prevalence of LA-MRSA within a herd, it still needs to be supplemented by other preventive or intervention measures.

The assumption regarding PS has a noticeable influence on the results. Effects might differ between different farm types contaminated with LA-MRSA at different levels and this simulation study highlights a strong need for more knowledge from on-farm trials.

## Conclusions

Reducing the transmission rates after LA-MRSA had become fully established within a herd, resulted in a marked decrease in the prevalence, but LA-MRSA only rarely disappeared completely. So, while changes in antimicrobial consumption patterns might be a very important step towards reducing the prevalence of LA-MRSA within a herd, it still needs to be supplemented by other preventive or intervention measures.

Slightly reducing the number of pigs within each section, reducing mixing of pigs, or improving internal biosecurity after LA-MRSA had become established within the herd only resulted in marginal changes in the median prevalence within the herd. However, these factors might be important in situations where LA-MRSA has not become established within the herd, or in relation to being able to achieve or maintain a low level of antimicrobial consumption.

The results of the sensitivity analysis indicated that the assumptions regarding the existence of pigs persistently shedding MRSA have a noticeable influence on the model results. The prevalence was in general, highest within the farrowing unit, and lowest within the mating unit, and thus the farrowing unit might be the area with most potential for intervention.

## Supporting information

S1 Table Use of buffer sections in Danish pig herds. (PDF)

S1 Fig Reduced transmission: Development in the median prevalence after intervention. (PDF)

S2 Fig Reduced transmission: Prevalence in the stable units six years after introduction. (PDF)

S3 Fig Reduced density: Low and medium transmission. (PDF)

S4 Fig Reduced mixing: Low and medium transmission. (PDF)

S5 Fig Improved internal biosecurity: Low and medium transmission. (PDF)

S6 Fig Mean prevalence following changes in transmission within one stable unit at a time. (PDF)

S7 Fig Sensitivity analysis: Persistent shedders. (PDF)

S8 Fig Sensitivity analysis: Transmission on the day of birth. (PDF)

S9 Fig Sensitivity analysis: Transmission on the day of birth—Reduced mixing and increased biosecurity. (PDF)
