# Supplementary material for: Mechanistic modelling of interventions against spread of livestock-associated methicillin-resistant Staphylococcus aureus (LA-MRSA) within a Danish farrow-to-finish pig herd
Source: PLoS One. 2018 Jul 12;13(7):e0200563. doi: 10.1371/journal.pone.0200563 (PMC6042764; doi:10.1371/journal.pone.0200563)
Supplement: S1 Table — (PDF) [file pone.0200563.s001.pdf]

S1 Table. Use of buffer sections in Danish pig herds.

Unit	Buffer section present on the farm
**Weaner unit**	63%
**Finisher unit**	52%

These results originate from a questionnaire-based phone interview survey conducted among Danish farmers during August 30, 2016 – April 12, 2017, where 166 farmers participated.
