# Supplementary material for: Mechanistic modelling of interventions against spread of livestock-associated methicillin-resistant Staphylococcus aureus (LA-MRSA) within a Danish farrow-to-finish pig herd
Source: PLoS One. 2018 Jul 12;13(7):e0200563. doi: 10.1371/journal.pone.0200563 (PMC6042764; doi:10.1371/journal.pone.0200563)
Supplement: S1 Fig — (PDF) [file pone.0200563.s002.pdf]

S1 Fig. Reduced transmission: Development in the median prevalence after intervention

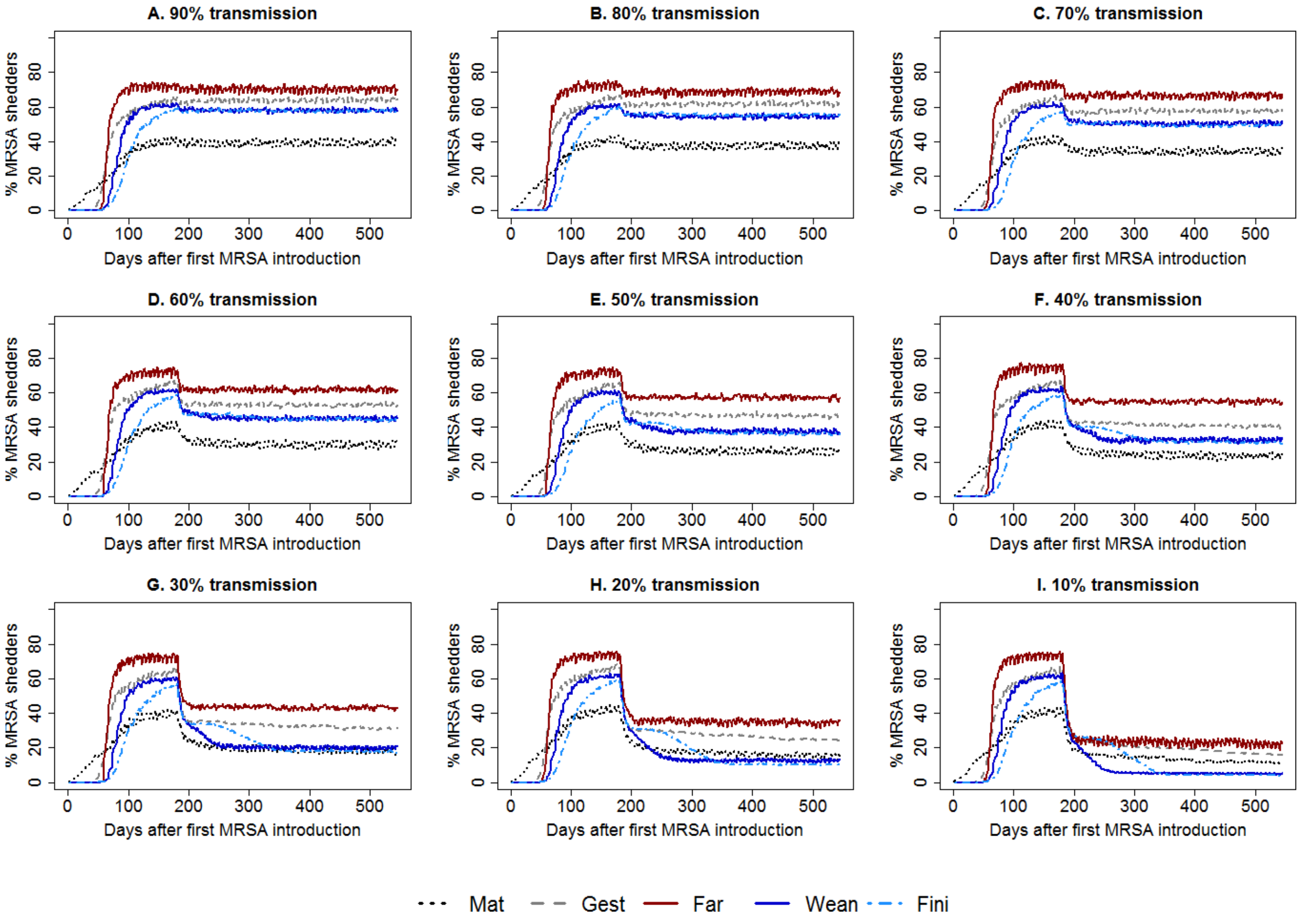

Note: Mat = mating unit, Gest = gestation unit, Far = farrowing unit, Wean = weaner unit, Fin = finisher unit. Transmission was reduced 180 days after MRSA had been introduced. The percentages refer to the proportion of the ‘high’ set of transmission rates, the set of rates used after 180 days corresponds to.
