# Supplementary material for: Mechanistic modelling of interventions against spread of livestock-associated methicillin-resistant Staphylococcus aureus (LA-MRSA) within a Danish farrow-to-finish pig herd
Source: PLoS One. 2018 Jul 12;13(7):e0200563. doi: 10.1371/journal.pone.0200563 (PMC6042764; doi:10.1371/journal.pone.0200563)
Supplement: S2 Fig — (PDF) [file pone.0200563.s003.pdf]

S2 Fig. Reduced transmission: prevalence in the stable units six years after introduction

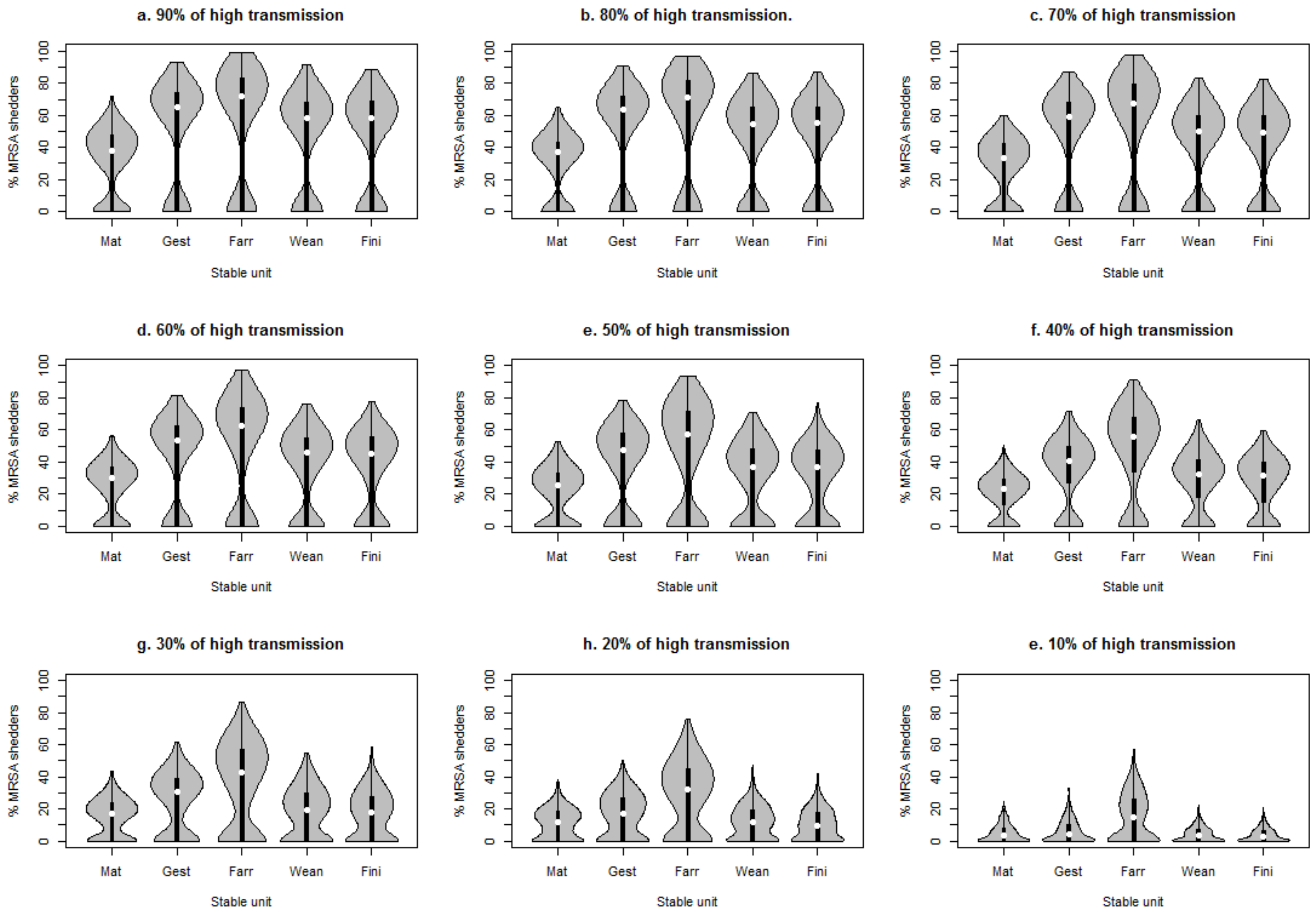

Note: Mat = mating unit, Gest = gestation unit, Far = farrowing unit, Wean = weaner unit, Fin = finisher unit. Mat = mating unit, Gest = Transmission was reduced 180 days after MRSA had been introduced.

The widths of the violin plots illustrate the distribution of 500 iterations. The median prevalence is indicated by white dots.
