# Supplementary material for: Mechanistic modelling of interventions against spread of livestock-associated methicillin-resistant Staphylococcus aureus (LA-MRSA) within a Danish farrow-to-finish pig herd
Source: PLoS One. 2018 Jul 12;13(7):e0200563. doi: 10.1371/journal.pone.0200563 (PMC6042764; doi:10.1371/journal.pone.0200563)
Supplement: S3 Fig — (PDF) [file pone.0200563.s004.pdf]

S3 Fig. Reduced density: Low and medium transmission

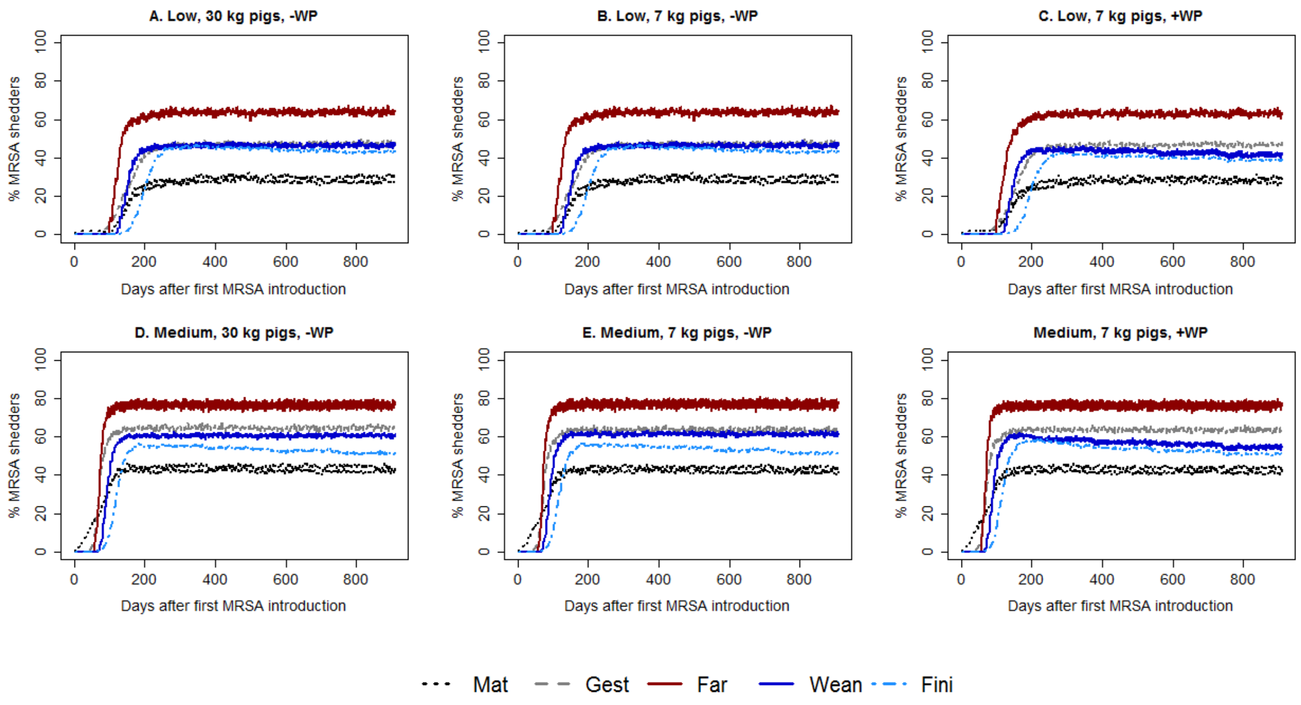

Note: Development in the median number and prevalence of MRSA shedders over time (only includes iterations where MRSA became established). Transmission was reduced 180 days after MRSA had been introduced.

Mat = mating unit, Gest = gestation unit, Far = farrowing unit, Wean = weaner unit, Fin = finisher unit.

7 kg pigs/30 kg pigs refer to if the pigs are sold just after weaning (7 kg) or not until they reach approximately 30 kg, which also is the point, where they normally would be moved from the weaner to the finisher unit.

-/+ WP (within-pen reduction refers) if the within-pen density has also been reduced, or if some pens are just empty; the overall within-room density will be the same in both scenarios.
