# Supplementary material for: Mechanistic modelling of interventions against spread of livestock-associated methicillin-resistant Staphylococcus aureus (LA-MRSA) within a Danish farrow-to-finish pig herd
Source: PLoS One. 2018 Jul 12;13(7):e0200563. doi: 10.1371/journal.pone.0200563 (PMC6042764; doi:10.1371/journal.pone.0200563)
Supplement: S4 Fig — (PDF) [file pone.0200563.s005.pdf]

S4 Fig. Reduced mixing: Medium and low transmission

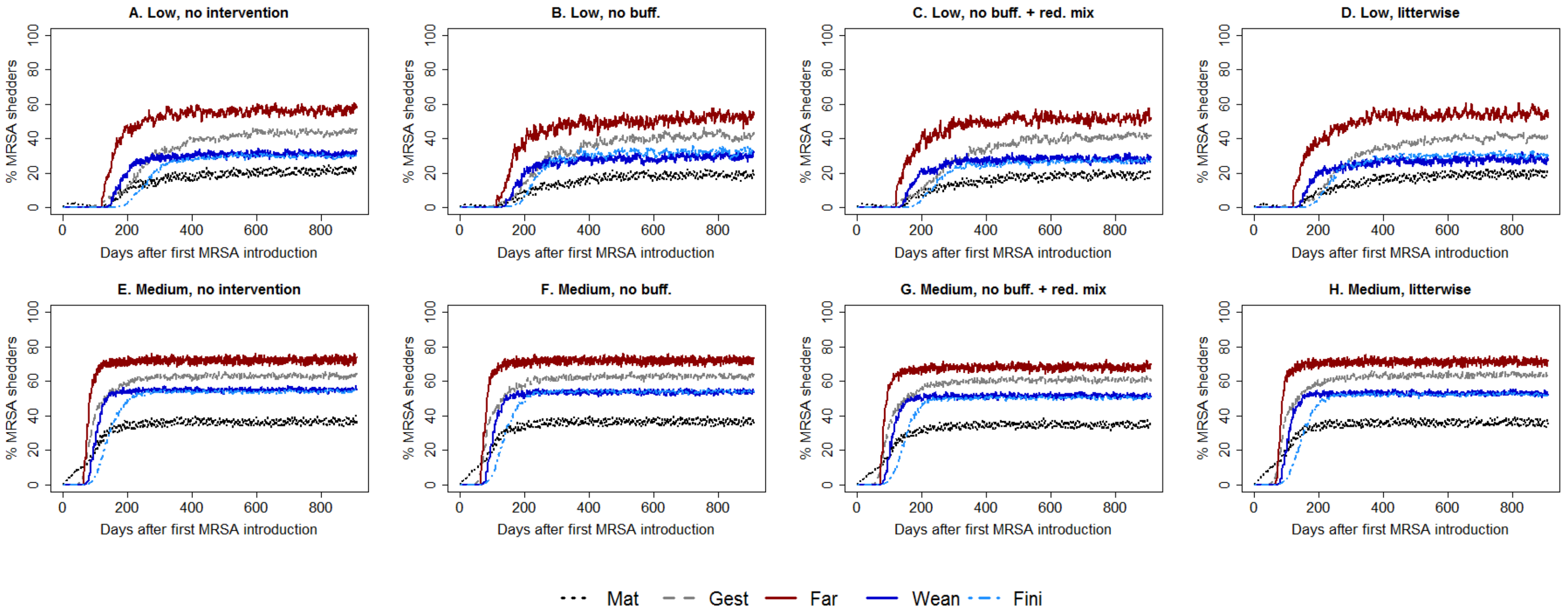

Note: Development in the median prevalence of MRSA shedders over time (includes only iterations where MRSA became established). Transmission was reduced 180 days after MRSA had been introduced.

Mat = mating unit, Gest = gestation unit, Far = farrowing unit, Wean = weaner unit, Fin = finisher unit.

No buff = no use of buffer sections, Red. Mix = Reduced mixing – two litters are put into one pen together in the weaners unit, instead of random mixing of piglets, Litterwise = weaners and finishers are only sharing pens with pigs from the same litter as themselves.
