# Supplementary material for: Mechanistic modelling of interventions against spread of livestock-associated methicillin-resistant Staphylococcus aureus (LA-MRSA) within a Danish farrow-to-finish pig herd
Source: PLoS One. 2018 Jul 12;13(7):e0200563. doi: 10.1371/journal.pone.0200563 (PMC6042764; doi:10.1371/journal.pone.0200563)
Supplement: S5 Fig — (PDF) [file pone.0200563.s006.pdf]

S5 Fig. Improved internal biosecurity: Low and medium transmission.

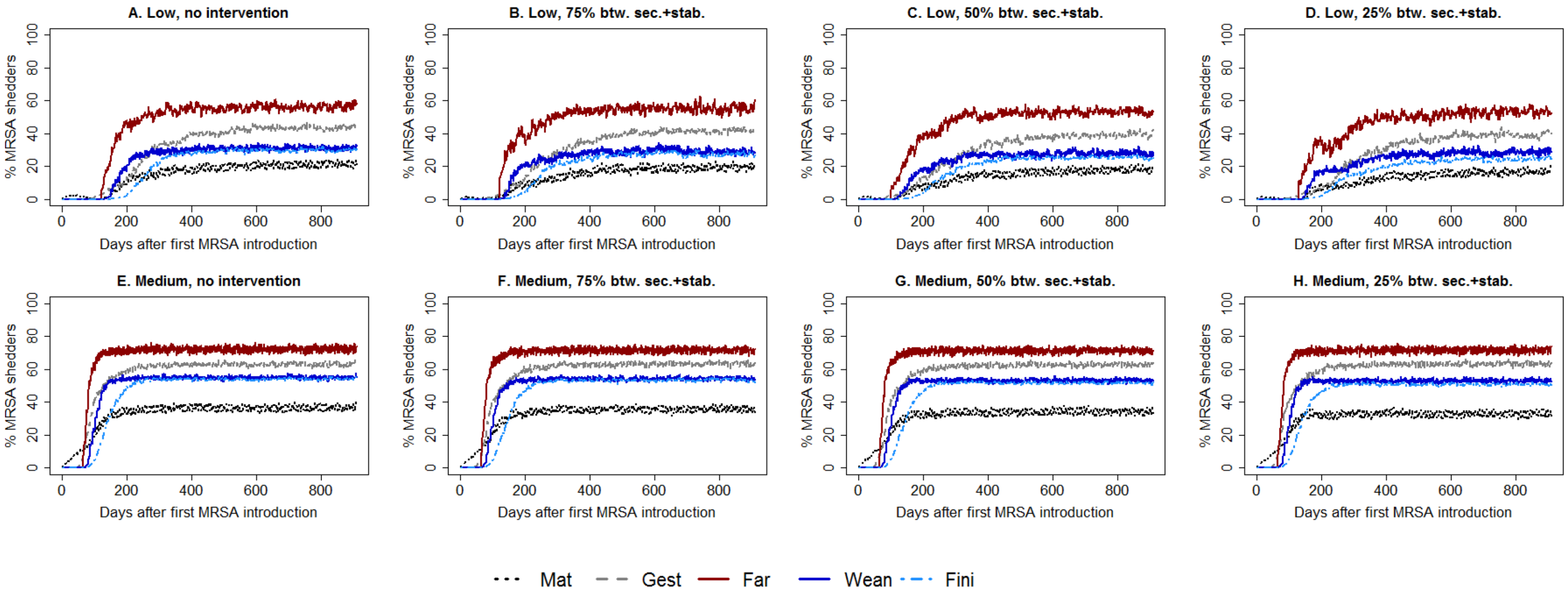

Note: Development in the median prevalence of MRSA shedders over time (includes only iterations where MRSA became established).
Mat = mating unit, Gest = gestation unit, Far = farrowing unit, Wean = weaner unit, Fin = finisher unit.
Transmission was reduced 180 days after MRSA had been introduced.
Panel B-D and F-H illustrate the influence of a gradual reduction of the transmission between sections and stables from 75% of the original value (B and F) to 25% of the original value (D and H).
