# Supplementary material for: Mechanistic modelling of interventions against spread of livestock-associated methicillin-resistant Staphylococcus aureus (LA-MRSA) within a Danish farrow-to-finish pig herd
Source: PLoS One. 2018 Jul 12;13(7):e0200563. doi: 10.1371/journal.pone.0200563 (PMC6042764; doi:10.1371/journal.pone.0200563)
Supplement: S6 Fig — (PDF) [file pone.0200563.s007.pdf]

**S6 Fig. Mean prevalence following changes in transmission within one stable unit at a time.**

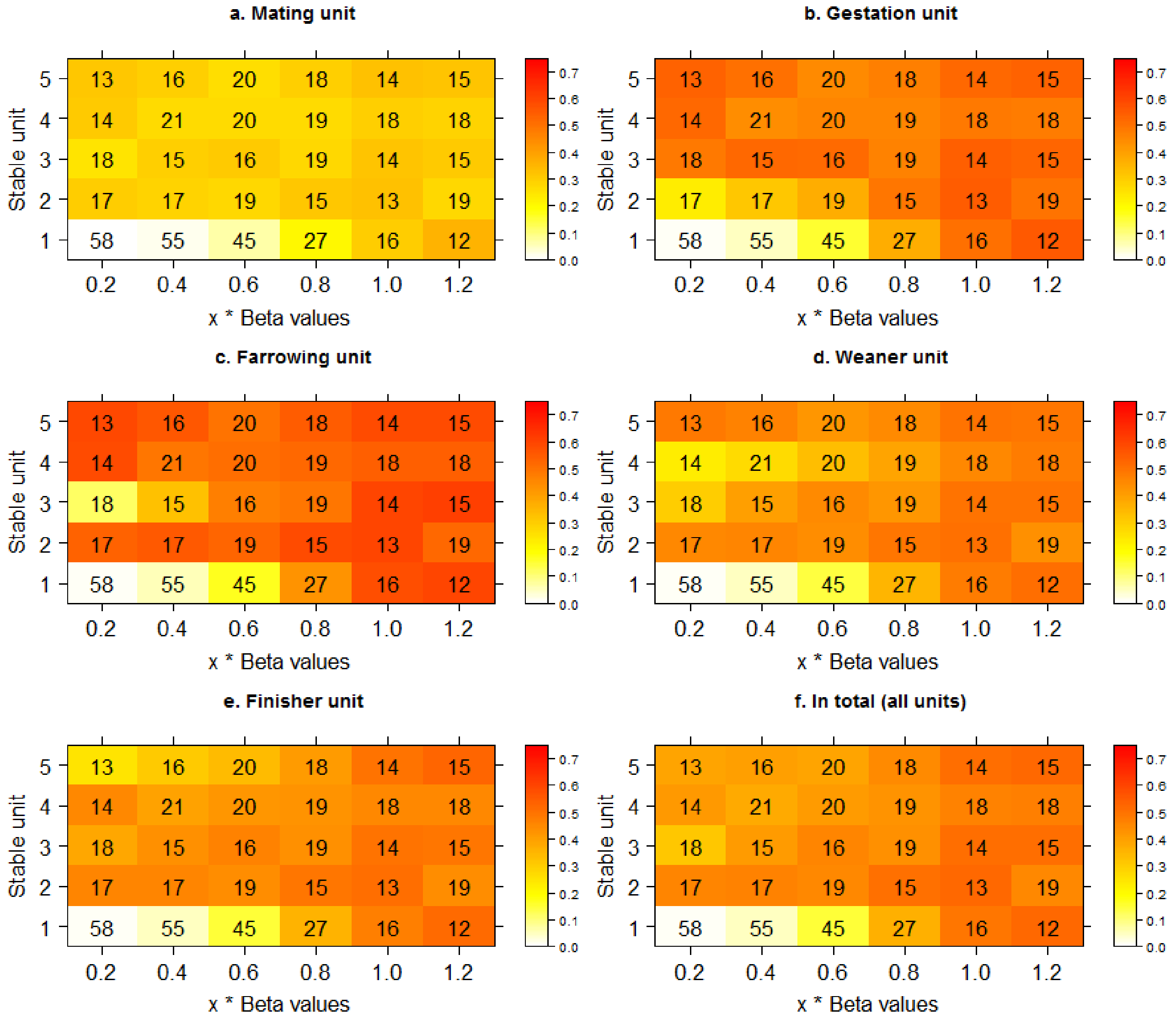


Note: The colour intensity represents the mean prevalence. The proportions of iterations where MRSA did not become established have been printed on each square. Stable units: 1. Mating unit, 2. Gestation unit, 3. Farrowing unit, 4. Weaner unit, 5. Finisher unit. x = proportion of the Beta values (transmission parameters) used. Beta values = all relevant transmission parameters applied within the unit.
