# Supplementary material for: Mechanistic modelling of interventions against spread of livestock-associated methicillin-resistant Staphylococcus aureus (LA-MRSA) within a Danish farrow-to-finish pig herd
Source: PLoS One. 2018 Jul 12;13(7):e0200563. doi: 10.1371/journal.pone.0200563 (PMC6042764; doi:10.1371/journal.pone.0200563)
Supplement: S7 Fig — (PDF) [file pone.0200563.s008.pdf]

S7 Fig. Sensitivity analysis: Persistent shedders.

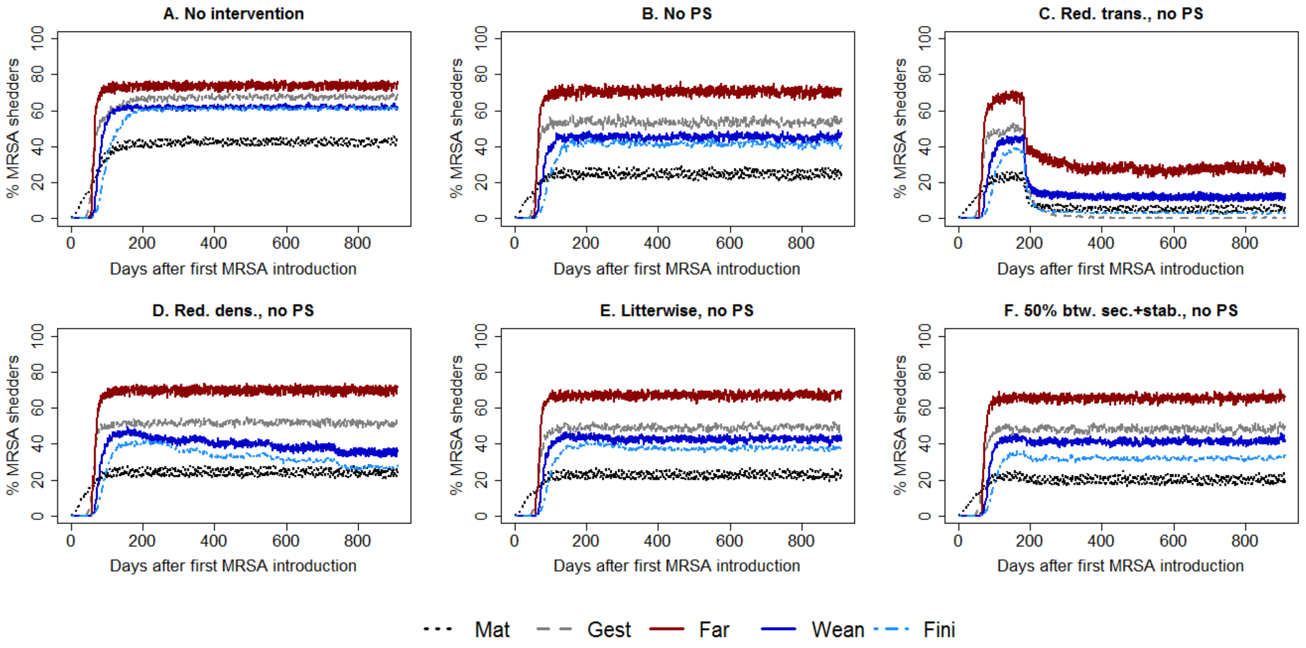

Note: Development in the median prevalence of MRSA shedders over time. High transmission.

No persistent shedders from the start of simulation. Transmission was reduced 180 days after MRSA had been introduced.

Mat = mating unit, Gest = gestation unit, Far = farrowing unit, Wean = weaner unit, Fin = finisher unit.

No PS = No existence of persistent shedders, it is assumed that all pigs will become intermittent shedders upon exposure.

Red. trans = transmission reduced to 40% of the initial level. Red. dens = sale of 7 kg pigs and increasing reduction in within pen density.

Litterwise = weaners and finishers are only sharing pens with pigs from the same litters as themselves.

50% btw. sec. + stab = the transmission between sections and stables reduced by 50%, e.g. through improved biosecurity.
