# Supplementary material for: Mechanistic modelling of interventions against spread of livestock-associated methicillin-resistant Staphylococcus aureus (LA-MRSA) within a Danish farrow-to-finish pig herd
Source: PLoS One. 2018 Jul 12;13(7):e0200563. doi: 10.1371/journal.pone.0200563 (PMC6042764; doi:10.1371/journal.pone.0200563)
Supplement: S8 Fig — (PDF) [file pone.0200563.s009.pdf]

S8 Fig. Sensitivity analysis: Transmission on the day of birth.

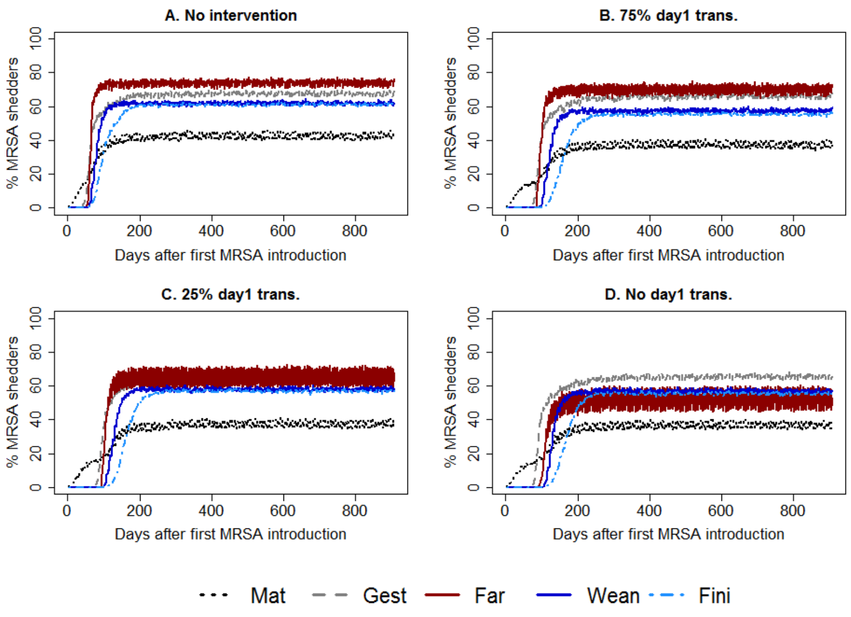

Note: Development in the median prevalence of MRSA shedders over time. High transmission and no intervention.
Mat = mating unit, Gest = gestation unit, Far = farrowing unit, Wean = weaner unit, Fin = finisher unit.
Panel A-D illustrate the influence of a gradual reduction of transmission between sow and offspring on the day of birth from 75% of the original value (B) to 0% of the original value (D).
